# Cellular population dynamics control the robustness of the stem cell niche

**DOI:** 10.1242/bio.013714

**Published:** 2015-10-09

**Authors:** Adam L. MacLean, Paul D. W. Kirk, Michael P. H. Stumpf

**Affiliations:** 1 Theoretical Systems Biology, Department of Life Sciences, Imperial College London, London SW7 2AZ, UK; 2 MRC Biostatistics Unit, Cambridge Institute of Public Health, Cambridge CB2 0SR, UK

**Keywords:** Stem cell niche, Cell fate, Self-renewal, Asymmetric division, Stability analysis

## Abstract

Within populations of cells, fate decisions are controlled by an indeterminate combination of cell-intrinsic and cell-extrinsic factors. In the case of stem cells, the stem cell niche is believed to maintain ‘stemness’ through communication and interactions between the stem cells and one or more other cell-types that contribute to the niche conditions. To investigate the robustness of cell fate decisions in the stem cell hierarchy and the role that the niche plays, we introduce simple mathematical models of stem and progenitor cells, their progeny and their interplay in the niche. These models capture the fundamental processes of proliferation and differentiation and allow us to consider alternative possibilities regarding how niche-mediated signalling feedback regulates the niche dynamics. Generalised stability analysis of these stem cell niche systems enables us to describe the stability properties of each model. We find that although the number of feasible states depends on the model, their probabilities of stability in general do not: stem cell–niche models are stable across a wide range of parameters. We demonstrate that niche-mediated feedback increases the number of stable steady states, and show how distinct cell states have distinct branching characteristics. The ecological feedback and interactions mediated by the stem cell niche thus lend (surprisingly) high levels of robustness to the stem and progenitor cell population dynamics. Furthermore, cell–cell interactions are sufficient for populations of stem cells and their progeny to achieve stability and maintain homeostasis. We show that the robustness of the niche – and hence of the stem cell pool in the niche – depends only weakly, if at all, on the complexity of the niche make-up: simple as well as complicated niche systems are capable of supporting robust and stable stem cell dynamics.

## Abstract (precis)

**Summary:** Stem cell niche dynamics are very robust to external and physiological perturbations because proliferation and differentiation are naturally balanced and controlled by the reliance on a shared niche environment.

## INTRODUCTION

Stem cells control the essential processes that facilitate multi-cellular life. Their ability to continue to produce more specialised types of cells in a coordinated manner underlies developmental processes, tissue regeneration and wound repair. Stem cell function relies crucially on the ability to make robust cell fate choices (Gurtner et al., 2008; Reya et al., 2001). These include the choice between self renewal and differentiation, or – once committed to differentiation – the choice between two or more specialised cell lineages. Many factors compound the decision-making process, ranging from cell-intrinsic regulation, to cell-extrinsic factors such as intercellular signalling and environmental stresses (Enver et al., 2009). Failure to make such cell fate choices correctly, by contrast, leads to disease and interferes with a host of physiological processes, ranging from control of the immune response to normal and healthy ageing (Brack et al., 2007; Geiger et al., 2013; Uccelli et al., 2008).

Stem cell function is therefore safeguarded by a number of mechanisms, including an apparently delicately balanced interplay with other cells. The concept of the stem cell niche (Schofield, 1978), has proved vital to our understanding of stem cell function and maintenance in a variety of cycling tissues including blood, skin and intestine (Reya et al., 2001; Spradling et al., 2001). Indeed, it can be argued that the ability of a cell to exhibit stemness cannot be defined in isolation, that is, without considering the influence of the niche. Conceptually, niches can be treated as domains of influence in which different cell populations can reside and exert effects on one another though signalling, paracrinal and juxtacrinal interactions. Such a description enables us to address questions regarding the particular extent, form, and constituents of a niche. Here, we focus of the question: what is the relationship between the complexity of the stem cell niche and its robustness? Despite significant overlap between niches as defined in stem cell biology and population biology (Mangel and Bonsall, 2013), few stem cell biologists have embraced the powerful interpretive framework offered by ecology. Here we show how a population biological perspective can guide our analysis and help to answer this (and other) questions.

Population biology has a rich history of applications, to systems ranging from ecological networks to social organisations (MacArthur and Wilson, 1967; MacLean et al., 2013; May, 1972; Nowell, 1976; Saavedra et al., 2011); the question whether such systems are robust or fragile has been central to many of these studies. Populations – whether they are composed of animal species or cell species – obey certain principles that both determine and are affected by the birth, growth, and death characteristics. These may be complex functions that depend on interactions with other species, which can be either positive (mutualistic), negative (competitive), or a mixture of the two. By integrating these processes we can build up a description of the dynamics of interacting populations. Stem cells and their progeny are well suited to such a description, thus, slowly, population biological concepts begin to gain a foothold in stem cell biology (MacLean et al., 2013; Székely et al., 2014).

Fixed points describe equilibria of a system: they are states at which population sizes remain stationary over time. If we take the blood system as an example, then a set of production and degradation rates that leads to constant population sizes for all cell species describes a fixed point of the system. Further analysis will be required in order to understand what happens to this system following a perturbation (such as bleeding or infection) from the fixed point.

The (local) stability of a population dynamical system refers to its ability to return to its initial state following a small perturbation away from a fixed point (Strogatz, 1994). An example of such stability is given by the return to homeostasis following a loss of red blood cells due to bleeding. Stability analysis enables us to measure the robustness of a particular state, and to ask which states are capable of persisting in nature. There has been much debate regarding how changes to the complexity of a system affect its stability. Early work supported the hypothesis that stability increased with complexity (Elton, 1958; MacArthur, 1955), however May subsequently proved a theorem stating that, in general, the stability of a system will decrease as complexity increases (May, 1972). More recent results have extended May's result and suggested that the system stability can change dramatically when specific types of interaction are considered (Allesina and Tang, 2012; Kirk et al., 2015). In particular, Kirk et al. (2015) relax the assumption that species only interact with one another at random, and in doing so move closer to a description of systems that we find in the natural world.

In order to investigate the stability of stem cell states, we develop models describing the dynamics of a stem cell lineage and study their equilibrium states – which vary between models – in order to determine which states can persist in nature. Since the stability of each state depends upon the values taken by the model's parameters, it is necessary to consider a range of biologically feasible parameter values. Building on previous work, we define the stability probability for a given state as the proportion of times that the state was found to be stable after repeatedly sampling parameter values from within this range (Christianou and Kokkoris, 2008; Gilpin, 1975; Kirk et al., 2015; Pimm, 1984; Roberts, 1974).

Here we are particularly interested in how the crucial stem cell processes of self renewal, differentiation and lineage choice affect the stability probability. These processes are regulated by feedback mediated by the niche. The form of this feedback is crucial, yet remains unknown; given the current difficulties of measuring niche dynamics *in vivo*, taking a modelling approach here presents significant advantages. We can, for example, decouple the effects on stem cells of signals from immature (progenitor) or mature, differentiated cells, and study each in turn.

We introduce a set of population dynamics models that share core attributes but differ in their number of lineages and feedback characteristics. The models chosen cover the relevant basic population scenarios: models with competition between cell types that are on different levels of the cell differentiation hierarchy; and models where intra- and inter-hierarchical competition is present, i.e. models where the hierarchy has two or more branches. Such ‘branched’ models represent a departure from traditional ecological systems, where potentially conflicting signals can arise from the progeny of a single population, and have been the subject of recent investigation regarding stem cell behaviours (Buzi et al., 2015). How the stability properties of branched (stem cell) systems depend on this competition is investigated here. Within these model scenarios we go on to consider different types of feedbacks between different cell-populations, where, e.g. the progenitor pools interact with the stem cell pool so that the latter can compensate for depletion of the former. More complicated or more realistic differentiation cascades are simply elaborations of the baseline scenarios exhibited here and lessons learned are straightforwardly transferred (as is shown in the Supplementary Information).

Structure within the models considered here (but also in any real-world systems) is a key factor in conferring and maintaining stability. Further analysis of one of the models demonstrates how different stable states can be reached from different experimental conditions (corresponding to different parameter values). This provides insight into how stem cells maintain homeostasis and how multiple states can be accessed, and could explain how, for example, depletion of a particular blood cell population is remedied at the stem/progenitor cell level by a state shift to one that repopulates the haematopoietic system.

## RESULTS

We analyse a set of representative models of the dynamics within the stem cell niche – we are particularly concerned with the haematopoietic stem cell niche but the analysis here will to a large extent carry through to other niches as well. We use simple population dynamics models that capture the expected behaviour of the occupants of a niche. While this model is simpler than a model that captures stochastic and spatial effects, our modelling framework has been shown to be in good agreement with more detailed agent-based modelling approaches.

We consider four exemplar models of within-niche dynamics out of the many different models that can be considered. Models of the stem cell niche have to account for the fact that progenitor cells, *P*, are derived from stem cells, *S*, and differentiated cells, *D* from progenitors (if we simplify the hierarchy typically observed in stem cells); in classical ecology species are in competition and never ‘produced’ from each other. We discuss one model defined by *S*→*P*→*D*, and three models where the stem cells give rise to two different progenitor cells P_1_ and P_2_ which then differentiate further. This allows us to consider cases where the different cell types in the niche are not direct descendants and where competition for niche resources is with cells at the same as well as at different levels of the differentiation hierarchy.

In the Supplementary Information we show that the results discussed below are characteristic for other models, too. Based on this, and recent developments in the stability analysis of dynamical/ecological systems, we can be certain that the findings reported here are generic features of models of the stem cell niche.

### Fixed points in the stem cell hierarchy define stable cell states

**Fig. 1.**
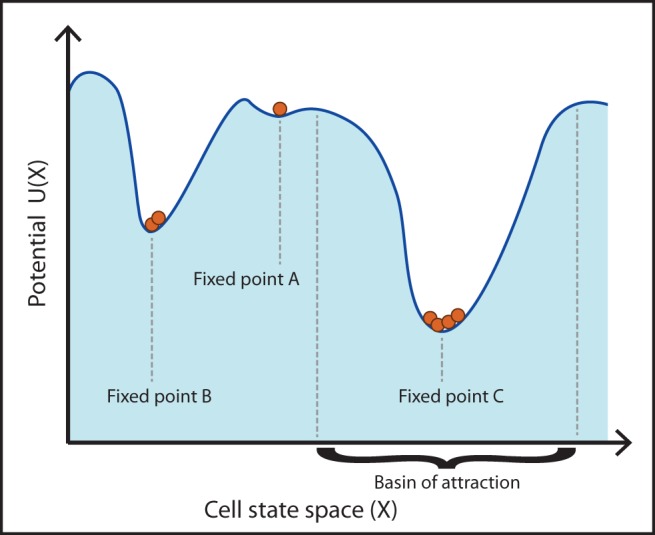
**Fixed points in cell state space.** Fixed points occur at minima on the landscape of cellular states, and correspond to persistent phenotypes. Each fixed point has a basin of attraction that defines the extent of its reach. Here, fixed point A may correspond to a stem or progenitor cell state, and fixed points B and C (with lower energy minima) to terminally differentiated cell states.

In order to assess the stability of cell states, we study the fixed points of model systems. Fixed points correspond to invariant states that are reached as a system approaches stationarity (other stationary states – such as oscillations or limit cycles – are also possible). In Fig. 1 we give an illustration of fixed points: these are the minima of the state space defined by a potential function, and cells lying within a basin of attraction will evolve towards them. Lower minima may correspond to terminally differentiated cell states, and higher minima – with higher potential – to stem or progenitor cell states. They can also be thought of as the local (or global) minima in Waddington's landscapes (Waddington, 1957) – but here the landscape corresponds to the population dynamics, and not the intra-cellular dynamics of stem cell behaviour as characterised by (e.g.) stem cell markers Nanog and Pecam for embryonic stem cells, or CD34 and Sca1 for haematopoietic stem cells (Rué and Martinez-Arias, 2015).

**Fig. 2.**
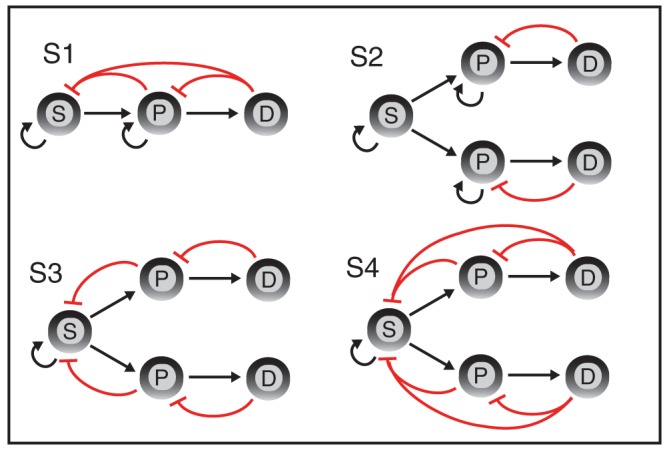
**Schematic description of models *S*1−*S*4.** Black arrows denote production by proliferation or differentiation and red arrows denote inhibition of cell proliferation by negative feedback. P, progenitor cells; S, stem cells; D, differentiated cells.

We consider typical, albeit simplified, stem cell differentiation hierarchies, consisting of three cell populations: stem cells (*S*), progenitor cells (*P*), and differentiated cells (*D*). Four models are constructed from these cell populations, shown in Fig. 2; these differ in their branching and feedback characteristics (Buzi et al., 2015). The models are, of course, (vastly) simplified descriptions of more complicated processes, however they serve our goal to compare characteristics, and as such can provide insight into basic mechanisms of stem cell function. The details of and assumptions underlying the models are given in the Methods. For the analysis of fixed points of a system, we have developed methods of generalised stability analysis that allow us to characterise the fixed points of stem cell models and assess their stability (Kirk et al., 2015). We provide a description of these methods and the statistical procedures that we use in Methods. The crucial concept derived from these methods is the stability probability of a fixed point. This defines the probability that a fixed point of a model will be stable, given that we know that the model parameters will lie within some range, but we do not know their values.

Shown in Table 1 are the stability probabilities for each fixed point of the models under investigation in column a. The number of fixed points differs between models *S*1−*S*4. Each model has the origin as a fixed point (fixed point 1); this corresponds to a state where all species go extinct. We do not analyse these points further since they are not of biological interest (some might still have interesting mathematical properties). In addition fixed point 2 for models *S*3 and *S*4 is not reachable; that is, the system will never end up in this state starting within the parameter ranges that we study. In previous work, Roberts (1974) referred to reachable fixed points as feasible. Here we will also leave aside the unreachable fixed points, and proceed to analyse those fixed points that are both reachable and nonzero. The initial conditions did not affect the fixed point reached for any of the models and parameter ranges studied here. Furthermore, we found that for each of the fixed points, the basins of attraction in state space did not overlap, thus precluding bistability under these conditions.

**Table 1.**
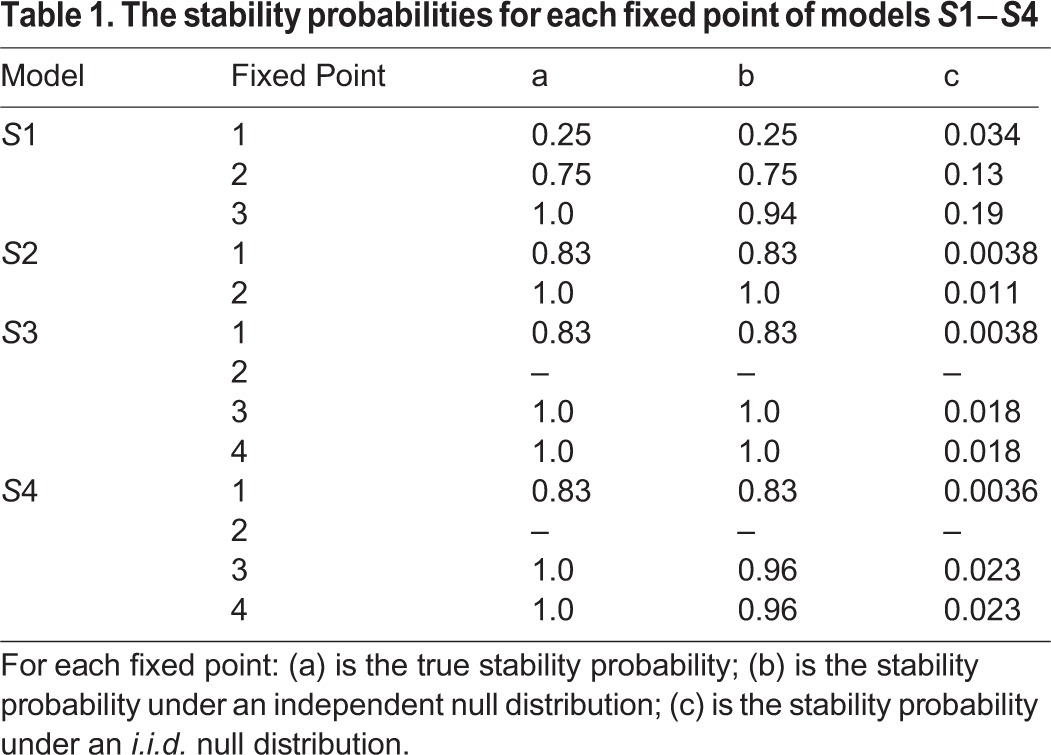
**The stability probabilities for each fixed point of models *S*1−*S*4**

The number of biologically meaningful (nonzero and reachable) cell states is two for models *S*1, *S*3, and *S*4, and one for model *S*2. We see that the number of cell types modelled does not correspond to the number of possible fixed points. For model *S*1 (see Fig. 2), fixed point 2 describes a state where progenitor and differentiated cell populations co-exist, but the stem cell pool has become completely depleted. The probability that this fixed point is stable is 0.75. The second fixed point describes a state where all three cell populations are positive, and this state will always be stable no matter where one begins in parameter space.

Of the three models that each represents five populations, model *S*2 has only one relevant fixed point – even fewer than model *S*1. This tells us that interactions only between differentiated and progenitor cells and not with the stem cell compartment limits the richness of dynamics available. The single biologically relevant state of model *S*2 is always stable. Model *S*3 has two reachable fixed points, both of which have positive population sizes for all five species (we now have branching in the stem cell compartment into two progenitor cell species). Each of these fixed points is stable for all parameter values: the system, by virtue of the nature of the cell–cell interactions is robust. For the final model, *S*4, we see that its stability properties closely reflect those of model *S*3: both biological fixed points (3 and 4) are always stable. Thus, the presence or absence of a direct signal from differentiated cells onto stem cells does not have great effect on cell state stability.

### Structure in the stem cell hierarchy maintains stability

In addition to the true stability probabilities obtained for each model state (Table 1, column *a*), we also calculate the stability probabilities under models that ignore the statistical dependencies inherent to real dynamical systems; this perspective has been very popular in population biology, where it was often (Allesina and Tang, 2012; May, 1972), but not always (Kirk et al., 2015; Roberts, 1974), seen as a valid attempt at assessing the stability of ecological systems. There the surprising result has been that large and complex ecological systems tend to be less stable than simple systems; the results in columns b and c in Table 1 correspond to the stability probabilities obtained for such models. See Methods for a description of how each of these distributions was calculated.

Upon comparison of columns a and c in Table 1, two main observations can be made. First, there are no significant differences between the stability probabilities given by the true and the independent distributions. This suggests that the dependencies between parameters of the system are not directly responsible for the stability of its cell state; rather it is the (feedback) structure of a differentiation cascade imposed by the niche microenvironment that confers stability. Second, there are striking differences between the stability probabilities for the true/independent and the *i.i.d.* distributions (see (Kirk et al., 2015) for further details). In most cases, the *i.i.d.* probability of stability is close to zero: so the structure of the stem cell ecology is far from random. While the structure alone suffices to determine stability, the detailed parameters (e.g. those determining the rates of asymmetric division) will be under the influence of natural selection and will reflect, for example, the physiological requirements for certain numbers/volumes of cells of each given type in a healthy (generally homeostatic) system.

### Detailed analysis of the stable states of model *S*3

More can be learned about the ecology of stem cells and their progeny by investigating the dynamics of these models more closely. In particular it allows us to start to understand the roles of individual parameters on the behaviour of such systems. As insights are also easily transferable between models, we discuss one model in detail. We therefore consider the possible stable states of model *S*3 (the other models exhibit qualitatively the same behaviour) and investigate what initial states lead to the behaviours associated with each of its biologically relevant fixed points. We find that although two states can be reached that yield positive population sizes for all species, bistability was not observed. This means that there do not exist any experimental conditions within the observed range from which both of the stable states can be reached; rather depending on the system parameters either one or the other will be attained.

We proceed to look at what differences there are in the distributions of parameters leading to each stationary state. From a total sample of 100,000 parameter sets, we find that approximately 8000 lead to fixed point 1 and another approximately 8000 lead to fixed point 2. It is interesting to note that only for this small proportion (16%) of possible parameter combinations states is it possible to reach biologically relevant states; the majority of parameters lead to extinction of species.

In order to ascribe significance to the results we obtain, we need to understand what state each of the fixed points corresponds to. Recall Fig. 2 for a graphical depiction of model *S*3: a stem cell gives rise to two progenitor cell populations, which here we call *A* and *B*. Each progenitor cell population can proliferate or differentiate into a corresponding differentiated cell population.

**Fig. 3.**
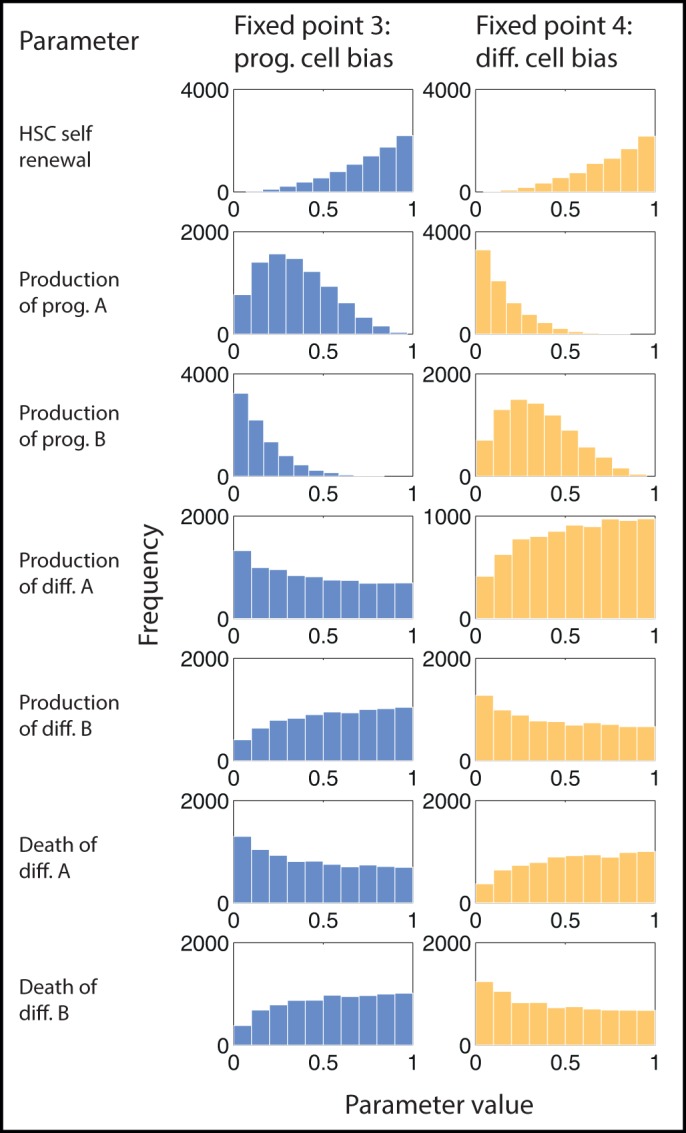
**Branching characteristics of distinct lineages.** Production rates of progenitor and differentiated cells affect the steady state reached. Histograms show the frequency of parameter values that lead to one of two possible fixed points for model *S*3. The first fixed point is characterised by a higher proportion of progenitor cells (prog. cell bias) and the second fixed point is characterised by a higher proportion of differentiated cells (diff. cell bias). ‘A’ and ‘B’ denote the two possible lineages that stem cells give rise to. To reach the progenitor cell-biased state, lineage A is favoured, whereas to reach the differentiated cell-biased state, lineage B is favoured.

Fixed point 3 corresponds to higher population sizes for progenitor cells by a factor of 10 – for both lineages – compared with the differentiated cell populations. Fixed point 4, in contrast, is characterised by higher levels of differentiated cell populations, again by approximately a factor of 10, relative to the progenitor cell populations. In Fig. 3 the distributions of parameters that give rise to these two different states are plotted, along with a description of their meaning.

By studying Fig. 3 we can describe the differences that lead to one fixed point or the other. To reach the state dominated by progenitor cells requires higher production rates of lineage *A* progenitors than lineage *B*. It also requires lower production and death rates of differentiated cells of lineage *A* compared to lineage *B*. We observe symmetries between the distributions that lead to each state: to reach the state dominated by differentiated cells requires, conversely, a higher production rate for progenitor cells of lineage *B* than lineage *A*, and lower production and death rates for the differentiated cells of lineage *B* than lineage *A*.

Describing how lineage bias influences the proportions of different cell species at steady state is especially interesting given the importance of branching fates in stem cell hierarchies, for example in the haematopoietic lineage between myeloid and lymphoid cell fates. The analysis performed here on fixed points 1 and 2 extends the concept of mapping a model's basin of attraction in parameter space. We can find what regions in parameter space lead to one, or another, fixed point and begin to delineate a boundary between them. Characterising the behaviour of a model with respect to a broad region in parameter space, rather than only at some specific values, enhances our understanding of a model and its potential use.

## DISCUSSION

The ability to make robust fate decisions in a stochastic environment, and the ability to remain a homeostatic population of differentiated, differentiating and stem cells, despite frequently low numbers in the stem cell compartment, is a characteristic feature of multi-cellular organisms (Garcia-Ojalvo and Martinez-Arias, 2012; Pauklin and Vallier, 2013; Philpott and Winton, 2014). Such a system can be disrupted, for example, by introducing cells with uncontrolled differentiation and proliferation patterns, i.e. cancer (akin here to an invasive species in classical ecology); then a set of new population dynamics takes over and determines the fate of the cell population. Quite generally, stem cells and their progeny represent populations of interacting cell species, analogous to populations of interacting species in ecology, and are thus amenable to being modelled using concepts from population biology. Substantial research has already been undertaken in ecology and has considered, in particular, the relationship between complexity and stability (Allesina and Tang, 2012; Elton, 1958; Ives and Carpenter, 2007; MacArthur, 1955; May, 1972; Roberts, 1974; Saavedra et al., 2011).

Mathematical analyses allow us to address questions that are inaccessible experimentally. Here we have assessed the stability properties of stem cell population models, focussing on the effects of heterogeneity and of dependencies between species in a hierarchy. Although we have increasing experimental power, for example, to image haematopoietic stem cells *in vivo* in the bone marrow (Lo Celso et al., 2009), or to study cancer in intestinal crypts (Drost et al., 2015), many stem cell processes are still not directly accessible to observation, and mathematical models can be used to link observables to underlying processes in a rational and hypothesis-driven way. Here the structure of the cell population – stem cells and their progeny – is found to be crucial in enabling stem cell systems (i.e. stem cells, progenitors and their descendants) to reach stable states; homogeneous (randomly interacting) cell populations are not stable. Parametric dependencies affect the stability to a much lesser extent, and we still find stable conditions for stem cells and their progeny to exist in homeostasis when the detailed parameters are ignored.

In order to investigate the population dynamics of stem cell systems, we have forsaken description of the underlying stochastic processes that govern cellular decision-making. Models that include stochasticity, either at the level of cell or molecular dynamics, can address questions regarding (e.g.) the effects of variability on stem cell robustness, and some progress has been made in such directions (Huang, 2010; Lei et al., 2014; Roeder et al., 2005). These models are appealing and offer much potential, but they are at best complementary to deterministic analyses, for which a host of results and analytical tools are available, enabling a level of model characterisation that is not possible for counterpart stochastic analyses.

We analysed the fixed points of one system (model *S*3) in more detail, as we found that multiple cell states could be reached by varying the *in silico* experimental conditions. The balance of progenitor and differentiated cells in model *S*3 is controlled by the propensity of stem cells to favour production of progenitor and differentiated cells in one of two possible lineages. Given the number of possible branching points in adult stem cell hierarchies, characterised by (for example) the multiplicity of haematopoietic progenitor cell species and the possible interactions between them (Wang and Wagers, 2011; Wilson and Trumpp, 2006), such asymmetries are very interesting to identify, and could exert key control over cell fate choice. To analyse this branching process further, comparison of this model to cell species data is required; this will allow us to distinguish between the model's two lineages.

The number of states and stability properties given by models *S*2 and *S*3 differ, however model *S*4 shares very similar fixed point characteristics to model *S*3. This suggests that whereas niche-mediated feedback onto stem cells is a key factor in state determination, whether the signal comes from the progenitor or the differentiated cell pool (the distinction between models *S*3 and *S*4) is much less important. The fact that structurally different (though related) models share qualitative features is encouraging as this suggests that such feedback might be a generic design feature shared across stem cell systems (Babtie et al., 2014). In light of the results and taken against the background of a vast body of work in population biology, it is certainly hard to propose other mechanisms that would confer such stability.

The definition of stability used throughout this work – that a system at a fixed point will return to the same fixed point following a small perturbation – at times may not match the biologically ‘stable’ properties that we aim to describe. One example of such a mismatch is that of oscillating systems, which can be stable in the sense of persistence. Another example is more subtle: if we compare two bistable systems, one where both fixed points have positive values for all species, and the other where for one fixed point at least one (or more species) goes to 0, we might wish to distinguish between them. That is, we might wish to call the perturbation from one state to another that causes at least one species to vanish greater (in the sense of being destabilising) than the perturbation that causes a state change that is not associated with the extinction of any species. This is an interesting avenue for future work where perhaps different criteria for stability could be used that reflect other aspects of biological homeostasis, such as species' extinction (Saavedra et al., 2011) or the effects of neutral mutations (Traulsen et al., 2012).

Recent theoretical and experimental studies suggest that multistability plays an important role in cell fate determination, demonstrated via studies of the Wnt signalling pathway (MacLean et al., 2015; Schuijers et al., 2015). While the bistable model of (MacLean et al., 2015) was proved to have two stable states for certain parameter values, similar analysis has not yet to our knowledge been performed for the feedback mechanism proposed in (Schuijers et al., 2015). Generalised stability analysis could shed light on the bistable regime controlled by the Ascl2 gene that is activated by Wnt; a system amenable to modelling. Here, the ecological perspective is perhaps most intimately coupled with cellular and molecular processes, and we can begin to study the multi-level and multi-scale interplay between these different levels *in vitro*, *in vivo* and *in silico*.

## CONCLUSIONS

Here we have seen that structure (in the sense of an underlying interaction network) bestows stability on such systems. We have shown how the stability dramatically decreases when structure is removed. We found this to be the case for all of the stem cell models that we studied here. For models with multiple biological steady states, we identified how each could be reached and in doing so mapped out the basins of attraction in parameter space. This provides insight into how branching decisions in stem cell hierarchies can be made. What emerges from this analysis is the remarkable robustness of stem-cell systems: their stability following a perturbation is a result of cell–cell interactions. This robustness has two consequences: (i) it provides stem cells with a stable ecosystem in which they can fulfil their function in e.g. maintaining tissue homeostasis; (ii) the flip-side is that malfunctioning stem-cell systems – such as systems with additional competition with cancer (stem) cells – may also be robust to a similar extent (MacLean et al., 2015; Youssefpour et al., 2012). Note, however, that the robustness we discuss here in no way limits the ability of stem cells and their progeny to exhibit considerable levels of heterogeneity – this is possible independently of the population dynamics (Rué and Martinez-Arias, 2015). As our understanding of the structure of stem cell ecosystems (as well as ecosystems more generally) increases, we learn how to shape their fate. The growing awareness that cancer is an evolutionary/ecological disease (Frank, 2007) is now opening up promising new directions for therapeutic intervention.

## METHODS

### Model development

Four models are introduced, each consisting of stem (*S*), progenitor (*P*), and differentiated (*D*) cell populations. The first of the models (*S*1) has three cell populations; the remaining three (*S*2−*S*4) have five. These extra two populations correspond to a branching point in the differentiation hierarchy (for example, in haematopoiesis, into myeloid and lymphoid lineages).

We make the assumptions that (i) renewal is restricted to *S* and *P*; (ii) only *D* are depleted through death/migration; (iii) differentiation is irreversible; (iv) a cell can influence its parent/grandparent population via intercellular signalling. The models are depicted in Fig. 2, and full description of their composition including the equations that govern them is given in Supplementary Information.

### Generalised stability analysis

In order to assess the stability of cell states, we calculate the Jacobian/community matrix, for a given state of the system (set of parameter values). This allows us to determine whether or not the system is stable in this state. We repeat this procedure for a large number of parameter sets, sampled in the parameter space in an attempt to capture the global behaviour characteristics of the system. From this analysis, we determine the probability that each fixed point of a given model is stable. We compare these probabilities with those derived from a null distribution, obtained by permuting the connections between cell populations at random. To calculate the independent null distribution we sample with replacement the distribution over each entry in the Jacobian, maintaining the entry position. To calculate the *i.i.d.* distribution we again sample with replacement from the Jacobian, but we now pool entries from all positions, thus the distribution from which we are sampling is now *i.i.d.* (Kirk et al., 2015). Further details of the methods of statistical analysis are given in Supplementary Information.
